# OmpA-Like Proteins of* Porphyromonas gingivalis* Mediate Resistance to the Antimicrobial Peptide LL-37

**DOI:** 10.1155/2018/2068435

**Published:** 2018-12-27

**Authors:** Toshi Horie, Megumi Inomata, Takeshi Into

**Affiliations:** Department of Oral Microbiology, Division of Oral Infections and Health Sciences, Asahi University School of Dentistry, 1851 Hozumi, Mizuho, Gifu 501-0296, Japan

## Abstract

Subgingival bacteria are continually exposed to gingival crevicular fluids that are derived from serum, which contain various bactericidal agents. The periodontopathic bacterium* Porphyromonas gingivalis* has been demonstrated to possess a variety of abilities to resist bactericidal agents, due to which it is able to propagate in the subgingival environment. We previously demonstrated that the major surface glycoproteins of* P. gingivalis*—Pgm6 and Pgm7, also called outer membrane protein A-like proteins (OmpALPs)—mediate resistance to the bactericidal activity of human serum, but their precise role remains unknown. In this study, we investigated the sensitivity of the wild-type and Pgm6/Pgm7-deficient* P. gingivalis* strains toward major antimicrobial peptides in the oral cavity, human *β*-defensins (hBDs) 1-3, and human cathelicidin LL-37. hBDs showed a considerably weak bactericidal activity against both bacterial strains. LL-37 also showed a weak activity against the wild-type strain; however, it showed a significant activity against the Pgm6/Pgm7-deficient strain. In the Pgm6/Pgm7-deficient strain, LL-37 remarkably accumulated on the bacterial cell surface, which may result in the destruction of the outer membrane. Additionally, the bactericidal activity of hBDs against the Pgm6/Pgm7-deficient strain was found to be synergistically promoted in the presence of LL-37. Our results suggest that OmpALPs specifically protect* P. gingivalis* from the bactericidal activity of LL-37; thus,* P. gingivalis* may adeptly survive in LL-37-producing subgingival environments.

## 1. Introduction

*Porphyromonas gingivalis*, a gram-negative, anaerobic, and asaccharolytic bacterium, is known as one of the most important periodontogenic pathogens in humans [1]. In adults, this bacterium propagates within the subgingival flora, the proportion of which increases in correlation with the severity of periodontitis [2, 3].* P. gingivalis* possesses a variety of abilities to disrupt host defense mechanisms [4, 5] and changes the bacterial composition of subgingival flora [6], which, in turn, promotes development of periodontitis. Furthermore,* P. gingivalis* propagated in the subgingival flora disseminates hematogenously to induce heterotopic infections, affecting cardiovascular diseases, Alzheimer's disease, and preterm birth [4, 7, 8].

Subgingival bacteria are continually exposed to gingival crevicular fluids that are derived from serum, which contain a wide variety of bactericidal agents [9, 10]. The propagation of* P. gingivalis* in such environments is dependent on its strong abilities to resist bactericidal agents. For example,* P. gingivalis* degrades complement components and antibodies by production of large-scale proteases [11, 12]. Furthermore,* P. gingivalis* recruits the complement inhibitor C4BP to the bacterial cell surface for inactivation of the complements [13] and produces capsular polysaccharide for surface protection [14]. Additionally, we recently demonstrated that the major surface glycoproteins of* P. gingivalis*—Pgm6 and Pgm7, also called outer membrane protein A- (OmpA-) like proteins (OmpALPs)—are crucial for resistance to the bactericidal activity of human serum, using mutant* P. gingivalis* strains that were deficient in the two genes encoding OmpALPs [15]. However, the precise mechanisms of OmpALP-mediated serum resistance have not been clarified yet.

The OmpALPs Pgm6 and Pgm7 are synthesized as* O*-linked glycoproteins [16] and are considered as transmembrane proteins that form heterotrimers or homotrimers [17]. We demonstrated the inability of the three OmpALP-deficient strains (a Pgm6-deficient strain, a Pgm7-deficient strain, and a Pgm6/7-double deficient strain) to grow in serum or serum-containing media, although they grow normally in the bacterial medium [15]. Similar to the wild-type strain, the OmpALP-deficient strains exhibited normal protease-producing activity, suggesting that the impaired serum resistance of the OmpALP-deficient strains does not involve protease synthesis. Moreover, the OmpALP-deficient strains cannot survive in heat-inactivated serum, but they gain the ability to grow in proteinase K-treated serum [15]. This suggests that OmpALPs can mediate resistance to bactericidal proteins or peptides excluding heat-labile proteins such as complements.

Antimicrobial peptides form a major part of the innate immune system in the oral cavity, including gingival crevicular fluids [9, 18, 19]. In particular, the cationic antimicrobial peptides human *β*-defensin (hBD) 1, hBD2, hBD3, and the human cathelicidin LL-37 peptide are important protective agents against infections of subgingival periodontogenic bacterial species, including* P. gingivalis* [9, 20, 21]. Moreover, cationic antimicrobial peptides do not induce resistance compared to traditional antimicrobial drugs [22]. In the present study, we therefore aimed to investigate the role of the OmpALPs of* P. gingivalis* in resistance to the bactericidal activity of these antimicrobial peptides.

## 2. Materials and Methods

### 2.1. Reagents

The antimicrobial peptides hBD1, hBD2, hBD3, and human LL-37 were obtained from the Peptide Institute (Osaka, Japan).

### 2.2. Bacterial Strains and Growth Conditions

*P. gingivalis* ATCC 33277 served as a wild-type strain. Three OmpALP-deficient (Pgm6-deficient, Pgm7-deficient, and Pgm6/Pgm7 double-deficient) mutant strains were produced by deleting* pg0695* and/or* pg0694* in the wild-type strain as described previously [15]. These strains were anaerobically grown in supplemented trypticase soy broth (sTSB) as described previously [15].

### 2.3. Detection of Bacterial ATP Production

Bacterial strains were cultured to the logarithmic phase in sTSB, and 1×10^7^ bacterial cells were suspended in 25 *μ*l of fresh sTSB followed by addition of 75 *μ*l of either blank phosphate-buffered saline (PBS) or PBS containing various concentrations of antimicrobial peptides. After anaerobic incubation for 6 h, bacterial survival was assessed by ATP production. Bacterial culture (100 *μ*l) and the reagent of BacTiter Glo microbial cell viability assay kit (Promega, Madison, WI, USA) (100 *μ*l) were mixed in wells of 96F Nontreated White Microwell SI plates (Thermo Fisher Scientific, Waltham, MA, USA). Bioluminescence was measured as relative light units (RLUs) using an Infinite M200 PRO plate reader (Tecan, Seestrasse, Switzerland). Bacterial survival (%) was calculated as 100×(experimental RLU)/(control RLU), where values for the control RLU were obtained from the bacterial culture added to blank PBS. Data are expressed as mean ± standard deviation (SD; n= 3). Representative results from more than three separate experiments are shown.

### 2.4. Bacterial Live/Dead Fluorescence Staining

Bacterial strains were anaerobically incubated in the presence of 5 *μ*M LL-37 for 24 h. The outer membrane integrity and viability of bacterial cells were assessed by a Bacteria Live/Dead staining Kit (Promokine, Heidelberg, Germany) that includes the green fluorescent stain DMAO for both live and dead bacterial cells and the red fluorescent stain Ethidium Homodimer-III (EthD-III) for dead bacterial cells. Staining was performed by incubating bacterial cells with the mixture of DMAO and EthD-III for 15 min. Stained cells were immediately imaged using a BZ-8000 fluorescence microscope (KEYENCE, Osaka, Japan).

### 2.5. Immunofluorescence for Bacterial Surface LL-37

Bacterial strains were anaerobically incubated in the presence of LL-37 (5 *μ*M) for 1 h. Bacterial cells were collected by centrifugation and washed twice with PBS followed by spotting on a coated slide glass for fixation with 4% paraformaldehyde for 10 min. Fixed cells were blocked with 3% BSA in PBS for 1 h. Cells were then stained with a mouse monoclonal antibody to LL-37 (sc-166770; Santa Cruz Biotechnology; Santa Cruz, CA, USA) and Alexa 488-conjugated anti-mouse IgG antibody (Thermo Fisher Scientific). Stained cells were embedded in the presence of the Prolong Gold Antifade reagent (Thermo Fisher Scientific). Images were captured using a BZ-8000 fluorescence microscope.

### 2.6. Statistical Analysis

**Figure 1:**
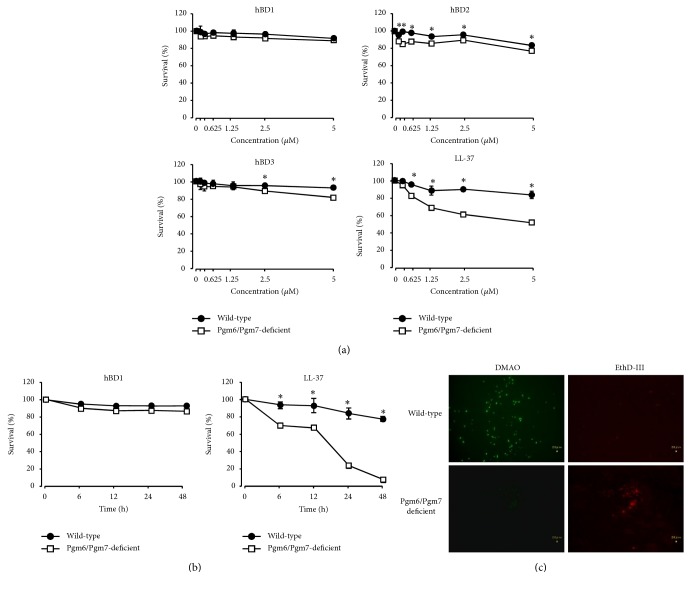
Sensitivity of the wild-type and OmpALP-deficient strains of* P. gingivalis* to the bactericidal activities of hBD1, hBD2, hBD3, and LL-37. (a) Bacterial cells (10^7^) of the wild-type and Pgm6/Pgm7-deficient strains, suspended in sTSB containing the 2-fold serial concentrations of the indicated antimicrobial peptides (0.156–5 *μ*M), were anaerobically cultured for 6 h. Bacterial survival was assessed by ATP production. Data are expressed as mean ± SD (n= 3). The statistical significance of differences was determined by the paired Student's* t*-test. *∗p* < 0.05. (b) Approximately 10^7^ wild-type and Pgm6/Pgm7-deficient bacterial cells were anaerobically cultured for the indicated periods (6–48 h) in the presence of hBD1 or LL-37 (5 *μ*M). Bacterial survival was assessed by ATP production. Data are expressed as mean ± SD (n= 3). The statistical significance of differences was determined by the paired Student's* t*-test. *∗p* < 0.05. (c) Approximately 10^7^ wild-type or Pgm6/Pgm7-deficient bacterial cells were anaerobically cultured for 24 h in the presence of LL-37 (5 *μ*M). The integrity of the outer membranes was assessed by fluorescence staining of bacterial cells with DMAO and EthD-III and then analyzed by fluorescence microscopy.

**Figure 2:**
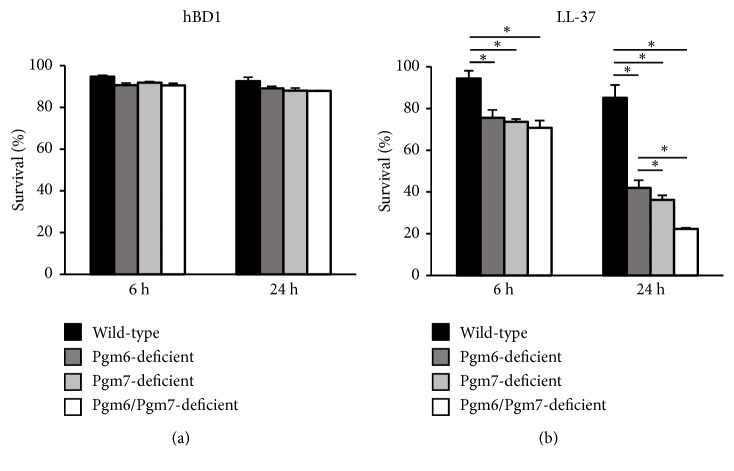
Sensitivity of the single OmpALP-deficient strains of* P. gingivalis* to the bactericidal activities of hBD1 and LL-37. (a, b) Approximately 10^7^ wild-type, Pgm6-deficient, Pgm7-deficient, or Pgm6/Pgm7-deficient bacterial cells were suspended in sTSB containing hBD1 or LL-37 (5 *μ*M) and anaerobically cultured for 6 and 24 h. Bacterial survival was assessed by ATP production. Data are expressed as mean ± SD (n= 3). *∗p* < 0.05, one-way ANOVA and Dunnett's test for post hoc comparisons (*μ*c≠*μ*i).

In the results of Figures 2 and 4, data were analyzed using two-way factorial analysis of variance (ANOVA) followed by Dunnett's multiple tests for comparison between the groups of interest. In the results of Figure 1,* p* values were calculated using Student's* t*-test. A two-tailed* p* value < 0.05 was considered significant.

## 3. Results

### 3.1. High Sensitivity of the OmpALP-Deficient* P. gingivalis* Strain to LL-37

We investigated the sensitivity of the wild-type and OmpALP-deficient strains to the antimicrobial cationic peptides hBD1, hBD2, hBD3, and LL-37. The growth of the* P. gingivalis* strains in the sTSB medium was previously confirmed to be identical [15]. Logarithmic-phase bacterial cultures of these strains were treated with the various concentrations of the antimicrobial peptides. The bacterial survival was assessed by measuring ATP production in the culture or by DMAO/EthD-III fluorescence staining of bacterial cells.

hBD1 hardly affected the survival of the wild-type and Pgm6/Pgm7-deficient strains at 0.156 – 5 *μ*M during the timeframe of 6 h (Figure 1(a)). hBD2 exhibited a slight bactericidal effect on the wild-type strain only at 5 *μ*M (Figure 1(a)). The bactericidal effect of hBD2 on the Pgm6/Pgm7-deficient strain was slightly stronger than that on the wild-type strain at 0.156–5 *μ*M. hBD3 hardly affected the survival of the wild-type strain at 0.156–5 *μ*M, but it lowered the survival of the Pgm6/Pgm7-deficient strain at 2.5 – 5 *μ*M (Figure 1(a)). LL-37 slightly affected the survival of the wild-type strain at 1.25 – 5 *μ*M (Figure 1(a)). On the other hand, LL-37 showed a remarkable bactericidal effect on the Pgm6/Pgm7-deficient strain at 0.625–5 *μ*M, which was significantly higher than that on the wild-type strain (Figure 1(a)). In the time-course study up to 48 h, hBD1 treatment at 5 *μ*M hardly affected the survival of the wild-type and Pgm6/Pgm7-deficient strains (Figure 1(b)). Of note, the Pgm6/Pgm7-deficient strain was almost dead after treatment with LL-37 at 5 *μ*M, whereas the wild-type strain was only slightly affected (Figure 1(b)). DMAO/EthD-III staining revealed that wild-type bacterial cells were almost intact. However, for the LL-37-treated Pgm6/Pgm7-deficient strain, the integrity of the outer membrane was completely lost, and dead cells were aggregated (Figure 1(c)).

We further investigated the sensitivity of the single mutant (Pgm6-deficient and Pgm7-deficient) strains to hBD1 and LL-37. The survival of the strains tested was hardly affected by hBD1 (Figure 2(a)). LL-37 significantly lowered the viability of all mutant strains, but the bactericidal activity of LL-37 was slightly reduced in the single mutant strains compared with that in the Pgm6/Pgm7 double-deficient strain (Figure 2(b)).

Thus, OmpALPs may serve as a protective factor for the outer membrane, by which the antimicrobial activity of LL-37 is impaired.

### 3.2. OmpALPs Protect* P. gingivalis* Cells by Preventing LL-37 Accumulation on the Cell Surface

**Figure 3:**
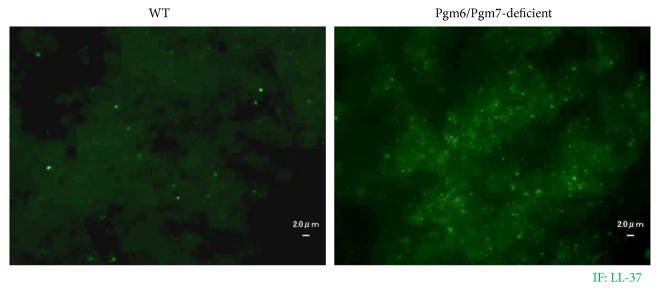
LL-37 on the cell surface was visualized by immunofluorescence staining in the LL-37-treated wild-type and OmpALP-deficient* P. gingivalis* cells. Approximately 10^7^ wild-type or Pgm6/Pgm7-deficient bacterial cells were treated with 5 *μ*M LL-37 for 1 h. The presence of LL-37 on the surface of fixed bacterial cells was visualized by immunofluorescence staining and analyzed by fluorescence microscopy.

We next investigated the mechanism by which OmpALPs protect* P. gingivalis* cells from the bactericidal attack of LL-37. The wild-type and Pgm6/Pgm7-deficient strains were treated with 5 *μ*M of LL-37 for 1 h, and then the presence of LL-37 was visualized by immunofluorescence staining. In the wild-type strain, LL-37 was hardly detected on the surface of bacterial cells, and a smaller number of LL-37 puncta was observed (Figure 3, left picture). On the contrary, in the Pgm6/Pgm7-deficient bacterial cells, LL-37 strongly accumulated on the surface (Figure 3, right picture). Thus, OmpALPs may have protected the* P. gingivalis* cells by inhibiting LL-37 accumulation on the cell surface.

### 3.3. Bactericidal Activity of hBDs against the OmpALP-Deficient* P. gingivalis* Strain Is Synergistically Promoted by LL-37

**Figure 4:**
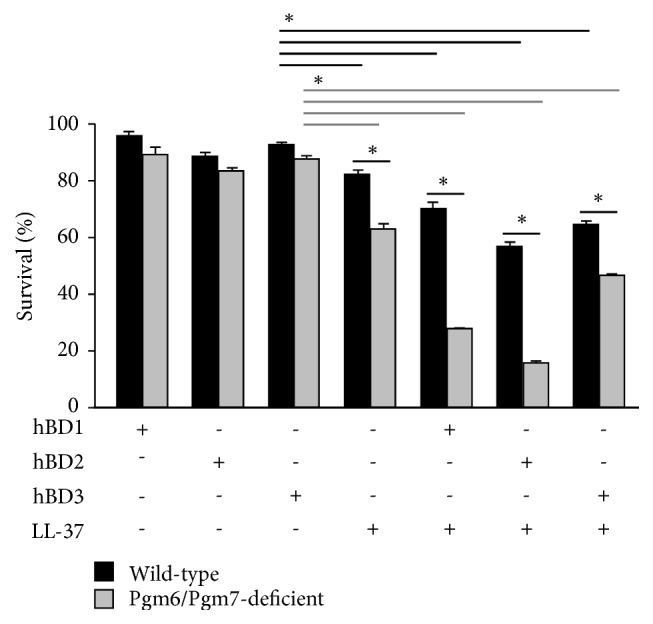
Sensitivity of the wild-type and Pgm6/Pgm7-deficient strains of* P. gingivalis* to the bactericidal activities of combinational treatment of hBD with LL-37. Bacterial cells (10^7^) of the wild-type and Pgm6/Pgm7-deficient strains, suspended in sTSB containing the indicated antimicrobial peptides (5 *μ*M), were anaerobically cultured for 6 h. Bacterial survival was assessed by ATP production. Data are expressed as mean ± SD (n= 3). *∗p* < 0.05, one-way ANOVA and Dunnett's test for post hoc comparisons (*μ*c≠*μ*i).

We further tested whether the combination of one of the hBDs with LL-37 could enhance the bactericidal activity. In the wild-type strain, although sole treatment of either hBD1, hBD2, hBD3, or LL-37, at 5 *μ*M, hardly showed a bactericidal effect consistent with Figure 1, the effect of LL-37 was slightly enhanced in combination with hBD, especially with hBD2 (Figure 4). In the Pgm6/Pgm7-deficient strain, consistent with Figure 1(a), sole treatment with LL-37 conferred a significant bactericidal effect, whereas hBD1, hBD2, and hBD3 did not confer a strong bactericidal effect (Figure 4). Of note, the effect of hBD, especially of hBD2, was synergistically enhanced in the presence of LL-37 (Figure 4). Thus, the Pgm6/Pgm7-deficient strain becomes sensitive to the bactericidal activity of LL-37, which probably enables hBDs to exert their bactericidal activities that are normally suppressed by OmpALPs.

## 4. Discussion

Our previous results indicated that OmpALPs play a crucial role in resistance towards certain bactericidal agents because the OmpALP-deficient strains could not survive in heat-inactivated serum, but possessed the ability to survive and grow in proteinase K-treated serum [15]. In the present study, we found that the OmpALP-deficient strain became sensitive against the bactericidal activity of LL-37. Moreover, although hBDs hardly exerted a satisfactory bactericidal effect on both the wild-type and OmpALP-deficient strains, they exerted a remarkable bactericidal effect in the presence of LL-37. Our results suggest that OmpALPs are important protective factors that defend* P. gingivalis* cells particularly from the bactericidal activity of LL-37. In the absence of OmpALPs, LL-37 accumulates on the* P. gingivalis* cell surface, which may result in the destruction of the cell surface, enabling other bactericidal agents to exert their activities, which are normally inhibited by OmpALPs. Thus, using OmpALPs,* P. gingivalis* may adeptly survive in gingival crevicular fluids and subgingival environments that contain LL-37 and other bactericidal agents, including hBDs.

All the antimicrobial peptides used in this study have a cationic property and are known to exert potent bactericidal activity against a wide range of bacterial species via a similar mechanism [23, 24]. LL-37 could exhibit a remarkable bactericidal activity against the OmpALP-deficient strain, but all hBDs exerted a weak bactericidal effect on* P. gingivalis* in the presence and the absence of OmpALPs. LL-37 is present as an amphipathic *α*-helical peptide, in which the hydrophilic amino acids form a positively charged side of the helix and the hydrophobic amino acids form the opposite negatively charged side of the helix [25]. The positively charged side interacts with the negatively charged hydrophilic head of phospholipids in bacterial cell membranes, whereas the hydrophobic side interacts with the hydrophobic lipid core of the bacterial membrane [23, 24]. In addition, the positively charged side of LL-37 interacts with the negatively charged moiety of lipopolysaccharides to neutralize their activity [26]. Thus, LL-37 inserts into the lipid bilayer of bacteria and perforates it, causing its breakdown into fragments [21]. Our results suggested that such a bactericidal activity of LL-37 can be potently inhibited by OmpALPs present on the surface of* P. gingivalis* cells. The exact mechanism of how OmpALPs affect LL-37 needs to be elucidated in a future study. In addition, it is important to explain why hBDs can exert a potent bactericidal effect against OmpALP-deficient* P. gingivalis* only in the presence of LL-37.

We compared the LL-37 resistance phenotype of the three OmpALP-deficient (Pgm6-deficient, Pgm7-deficient, and Pgm6/Pgm7 double-deficient) strains with that of the wild-type strain. The OmpALP proteins Pgm6 and Pgm7 have been reported to form the Pgm6/Pgm7 heterotrimers or Pgm7 homotrimer, but the Pgm6 homotrimers are prone to degradation [17], indicating that these two trimers were responsible for the function of OmpALPs. In the present study, we observed that the bactericidal activity of LL-37 was slightly reduced in the single mutant strains compared with that in the Pgm6/Pgm7-deficient strain. This result suggests that, in addition to the Pgm6/Pgm7 heterotrimer and Pgm7 homotrimer, the Pgm6 homotrimer may also be responsible for the resistance to LL-37. This may be inconsistent with a previous result indicating that the Pgm6 homotrimer was not functional [17].

The C-terminal region of OmpALPs, Pgm6 and Pgm7, is highly similar to the C-terminal region of* Escherichia coli* OmpA [27]. The N-terminal domain of OmpA has an eight-stranded structure and is thought to function as a porin [28], whereas OmpALPs have only one putative transmembrane region in the N-terminus that is hypothesized to not function as porins [17]. OmpA is highly conserved among bacteria of the Enterobacteriaceae family [28], whereas OmpALPs are conserved among bacteria of the Porphyromonadaceae family [15]. OmpA is known to participate in various pathogenic processes, including cell adhesion, cell invasion, intracellular survival, serum resistance, and inhibition of activity by bactericidal agents [28]. As we have demonstrated that OmpALPs are responsible for the serum resistance and inhibition of activity by bactericidal agents, OmpALPs and OmpA may share similar biological functions. It will be necessary to determine whether other bacterial species exhibit OmpALP- or OmpA-mediated resistance to the bactericidal activity of LL-37.

## 5. Conclusion

In conclusion, the major surface glycoproteins of* P. gingivalis* called OmpALPs were found to mediate resistance to the bactericidal activity of LL-37. Moreover, although hBDs hardly exerts a satisfactory bactericidal effect even on OmpALP-deficient* P. gingivalis*, they exert a remarkable bactericidal effect in the presence of LL-37. The future investigations could further reveal the specificity of the protective activity of OmpALPs for antimicrobial agents. Moreover, it should be also determined whether targeting the function or the production of OmpALPs leads to suppression of* P. gingivalis* infection or facilitates the treatment of chronic periodontitis.

## Data Availability

The data used to support the findings of this study are available from the corresponding author upon request.
